# BARRIERS TO VILLAGE MEMBERSHIP AMONG MINORITY SENIORS

**DOI:** 10.1093/geroni/igz038.840

**Published:** 2019-11-08

**Authors:** Carrie Graham, Winston Tseng

**Affiliations:** 1 University of California, Berkeley, California, United States; 2 University of California, Berkeley, Berkeley, California, United States

## Abstract

Villages are a relatively new consumer-driven model that promotes aging in place for community-dwelling seniors. Villages promote social engagement, civic engagement, member-to-member-support, and collectively bargain for services of their members. Members report improved social support and more confidence aging in their own homes. Currently, there are over 200 operational villages nationwide and the model is proliferating rapidly. Most Villages members are white, well-educated, and well resourced. Researchers at UC Berkeley conducted 6 focus groups with Latino, African American and Asian seniors (N=58) who have not joined Villages in their regions. Focus group findings describe a lack of awareness of the Village model among underrepresented groups; and barriers to membership including the cost of membership, lack of language inclusion, and lack of diversity. The national anti-immigrant discourse emerged as a barrier to membership for non-white seniors. Participants describe how Villages could make programmatic changes to attract a more diverse membership.

